# A complete chloroplast genome of *Keteleeria davidiana* (Pinaceae) and its phylogenetic implications

**DOI:** 10.1080/23802359.2021.1907253

**Published:** 2021-06-21

**Authors:** Min Zhang, Yuan-Yuan Li, Zi-Han Chai, Yue Zhu, Yi-Fan Duan

**Affiliations:** Co-Innovation Center for Sustainable Forestry in Southern China, College of Biology and the Environment, Nanjing Forestry University, Nanjing, PR China

**Keywords:** *Keteleeria davidiana*, chloroplast genome, taxonomic study

## Abstract

*Keteleeria davidiana* (Bertrand) Beissner 1891 (Pinaceae) is a rare tertiary relict plant endemic to China. However, since the main morphological characteristics used for identifying *K. davidiana* are variable, some taxonomic treatments within the species are still controversial. Here a complete chloroplast genome of *K. davidiana* representing a special genotype was assembled, which could provide more information for the taxonomic study of this species. The assembled genome was 117,642 bp in length with a large single-copy (LSC) region (74,825 bp), a small single-copy (SSC) region (40,247 bp), and two incomplete inverted repeats (IRs) regions (1285 bp each). In total, 118 genes were predicted, including 4 *rRNA*s, 34 *tRNA*s, and 80 protein-coding genes. The overall GC content of the assembled genome was 38.5%. Phylogenetic analysis showed that different accessions of *K. davidiana* formed a clade with relatively low support (bootstrap value = 71), which indicated a high level of sequences variation within the species.

*Keteleeria davidiana* (Bertrand) Beissner 1891 (Pinaceae) has long been treated as a rare tertiary relict plant endemic to China. However, since the main morphological characteristics used for identifying *K. davidiana* are variable, some taxonomic treatments within the species are still controversial. For example Cheng et al. (1975) published *K. pubescens* W. C. Cheng & L. K. Fu 1975, which was reduced as either a variety (Silba 1990) or a subspecies (Silba 2008) of *K. davidiana*, subsequently. In the latest literature (The Biodiversity Committee of Chinese Academy of Sciences 2020), *K. pubescens* was no longer recognized but treated as a synonym of *K. davidiana*. All these make the classification of *K. davidiana* extremely complicated. To address this issue, a complete chloroplast genome of *K. davidiana*, representing the genotype of ‘*K. pubescens*’, was assembled by using the next-generation sequencing, which could provide more information for the taxonomic study of this species.

The material of *K. davidiana* used in this study was collected from Kunming Institute of Botany, Chinese Academy of Sciences (25°8′36.91′′N, 102°45′8.46′′E). The voucher specimen was deposited at the herbarium of Nanjing Forestry University (Contact: Xian-Rong Wang, wangxianrong66@njfu.edu.cn) under the voucher number NF20200927002. Total DNA was isolated from the fresh leaves for sequencing library construction. Then the library was paired-end sequenced on the Illumina NovaSeq 6000 platform (Illumina Inc., San Diego, CA) by Genepioneer Biotechnologies Co., Ltd. (Nanjing, China) with the standard Illumina re-sequencing protocols. Finally, 6 Gb clean reads (Phred scores > 20) were obtained. The clean reads were first mapped to the plant chloroplast genome database using Bowtie2 (Langmead and Salzberg 2012) software for chloroplast reads extraction. Then SPAdes assembler version 3.10.0 (Bankevich et al. 2012) combined with SSPACE (Boetzer et al. 2011) was used for genome assembly. After that, GapFiller version 1.11 (Boetzer and Pirovano 2012) was further applied for gap filling, followed by PGA (Qu et al. 2019) software for genome annotation. The assembled chloroplast genome of *K. davidiana* was 117,642 bp in length with a large single-copy (LSC) region (74,825 bp), a small single-copy (SSC) region (40,247 bp), and a pair of inverted repeats (IRs) regions (1285 bp each). Unlike most angiosperm, the IR regions in *K. davidiana* were incomplete. Finally, 118 genes were predicted, including four *rRNA*s, 34 *tRNA*s (33 unique species), and 80 protein-coding genes (78 unique species). The overall GC content of the assembled genome was 38.5%.

Maximum likelihood (ML) tree was predicted to reveal the taxonomic status of *K. davidiana*. In total, 19 chloroplast genome sequences were used for phylogenetic tree construction including 18 downloaded from NCBI (Figure 1). PhyloSuite software (Zhang et al. 2020) was used for genic region extraction, and only genic regions were used for phylogenetic tree construction. The data matrix was aligned by MAFFT version 7.158 (Katoh and Standley 2013). Then RAxML-VI-HPC (Stamatakis 2006) was used for phylogenetic inference under the GTR-gamma model with a bootstrap test of 1000 replications. Finally, the ML tree was edited and visualized using Figtree software (http://tree.bio.ed.ac.uk/software/figtree/). As shown in Figure 1, two accessions of *K. davidiana* formed a clade with relatively low support (bootstrap value = 71). There were 80 single nucleotide polymorphisms (SNPs) and 15 insertion/deletion (indels) variations between the genome assembled in this study and the earlier one (Wu et al. 2009). Most of these variations (63 SNPs and 14 indels) were at non-coding regions. The sequence identity between the two chloroplast genomes was 99.93%, which indicated a high level of sequences variation within the species.

**Figure 1.**
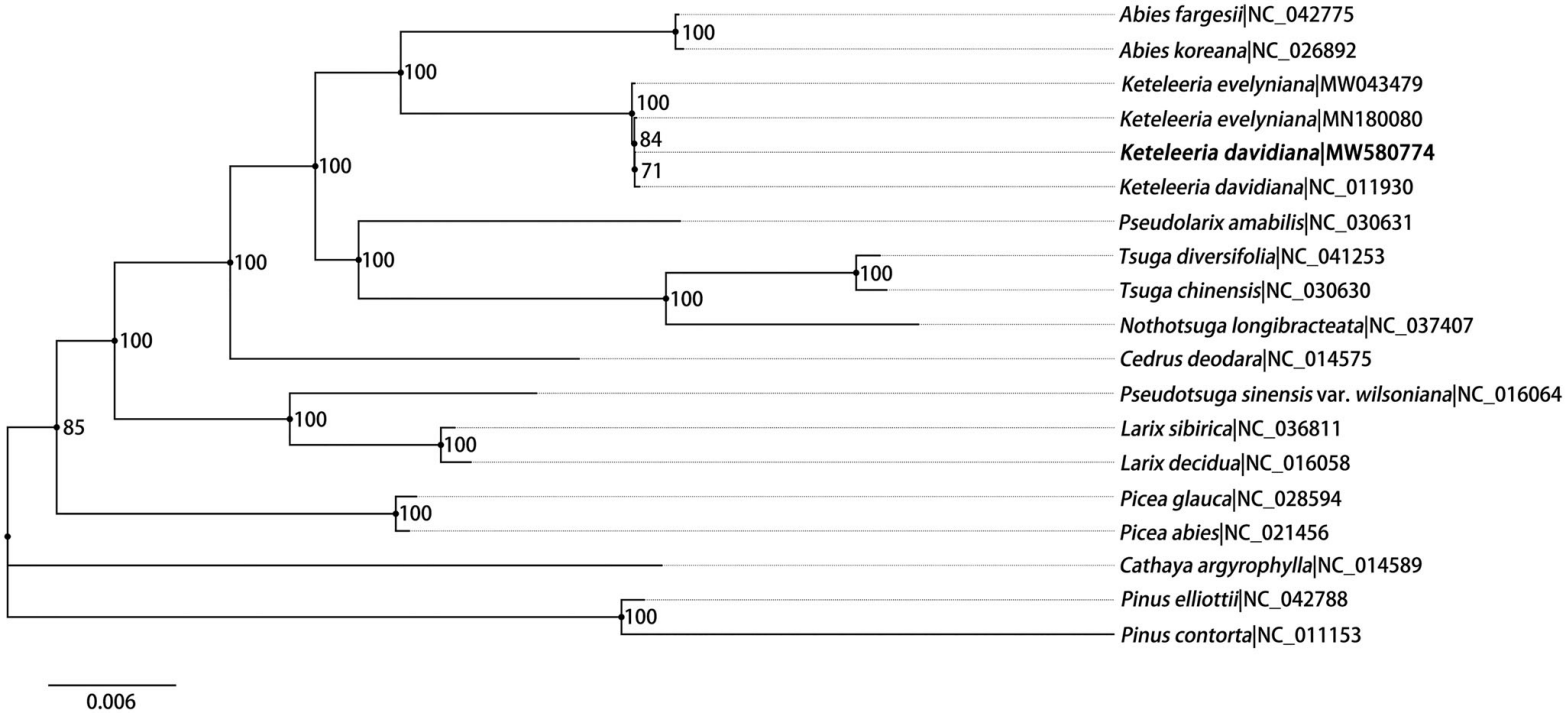
Maximum-likelihood phylogenetic tree based on 19 complete plastid genomes from Pinaceae. Two species from *Pinus* are set as outgroup. Number at the node shows bootstrap value (1000 replicates) for each branch. Number after ‘|’ shows the accession number in GenBank for each taxon. The position of *Keteleeria davidiana* reported in this study is marked in bold.

## Data Availability

The raw sequence data supporting this study are deposited in the National Center for Biotechnology Information Short Read Archive under BioProject ID PRJNA700108 (accession number SRP305000). The assembled genome and its annotation are openly available in GenBank of NCBI at https://www.ncbi.nlm.nih.gov, reference number MW580774.
